# Individual and Community Engagement in Response to Environmental Challenges Experienced in Four Low-Income Urban Neighborhoods

**DOI:** 10.3390/ijerph17061831

**Published:** 2020-03-12

**Authors:** Michelle L. Kaiser, Michelle D. Hand, Erica K. Pence

**Affiliations:** College of Social Work, Ohio State University, Columbus, OH 43222, USA; hand.49@buckeyemail.osu.edu (M.D.H.); pence.146@buckeyemail.osu.edu (E.K.P.)

**Keywords:** urban environment, pollution, eco-social, environmental justice, individual efficacy, community engagement, built environment, focus groups

## Abstract

Low-income urban communities, and the individuals that live within them, continue to face disproportionate interconnected social, economic, and environmental challenges related to their built, natural, and social environments. The aim of our phenomenological research study was to elevate the experiences of residents living in low-income urban neighborhoods in terms of their communities’ environmental challenges. Our objectives were to (1) identify challenges across neighborhoods, (2) identify ways individuals and communities are addressing those challenges, and (3) assess the individual and collective efficacy and engagement of communities to lead environmental improvements in neighborhoods. This study brings forward the voices that are often ignored or misunderstood in these communities and uses an ecological-social perspective. We conducted focus groups (N = 68) in four low-income urban neighborhoods across two Ohio cities in the United States. Participants described five key challenges in their communities: Pollution, abandoned buildings with associated crime, low food access and health concerns, trash and illegal dumping, and lack of trees. We assessed engagement and efficacy using two frameworks focused on individual and community readiness to engage in and lead community change. Policymakers should acknowledge the valuable contributions and leadership capacity of residents in low-income communities to implement environmental initiatives.

## 1. Introduction

Low-income urban communities, and the individuals that live within them, continue to face disproportionate interconnected social, economic, and environmental challenges related to their built, natural, and social environments [1,2,3,4,5]. Negative characteristics in the built environment (e.g., traffic, poor public transportation, lack of green space, concern for safety, dirtiness) can negatively impact the physical and mental health of individuals and the community [3,4,6,7,8,9]. In addition, economic and social characteristics (e.g., low median income, poverty, stress, crime rates, race, food access) in neighborhoods also contribute to poor health [3,10]. 

The aim of our phenomenological research study was to elevate the experiences of residents living in low-income urban neighborhoods in terms of the environmental challenges their communities were facing. Our objectives were to (1) identify challenges across neighborhoods, (2) identify ways individuals and communities are addressing those challenges, and (3) assess the individual and collective efficacy and engagement of communities to lead environmental improvements in neighborhoods. Our research moves beyond previous research focused more on residents’ descriptions of their environment. This research also provides an analysis of motivations, perceptions, and characteristics of individuals and communities who have been inspired to act or have the capacity to take action.

This study was conducted in four low-income neighborhoods across two cities in a Midwest state. Each neighborhood has its own unique history, with the land, the economy, and the people shifting and adapting to periods of rapid urbanization, economic recessions, downsized manufacturing industries, white flight, drug crises, red lining, flooding, disinvestment, revitalization, gentrification, and overall abandonment. We hosted four focus groups with individuals living in these spaces, as they reflected on their built, natural, and social environment. Our study brings forward the voices that are often ignored or misunderstood in these communities. We highlight the residents’ ideas and solutions for improving their neighborhoods. Policymakers should acknowledge these valuable contributions, ideas and leadership capacity of residents in low-income communities to implement environmental initiatives.

## 2. Background/Literature Review 

The social and physical environment has been shown to be as important of a cause of mortality as human behaviors and underlying biological mechanisms of disease [11]. Public health concerns in the United States related to the community environment include obesity and chronic diet-related diseases [12,13,14,15,16], respiratory diseases like asthma [6], and poor mental health (e.g., depression, anxiety) [7,17,18,19]. Though the mechanisms are complex and intertwined with race/ethnicity and socioeconomic status [3,11], poor housing conditions [1,6,20,21], environmental pollution (e.g., air, noise) [8,17,20,21,22,23], violence, and fear of violence [24,25] contribute to these public health outcomes. 

Research related to the interactions between humans and their social, natural, and built environments exists in a diverse range of academic and practice-oriented disciplines (e.g., social epidemiology, public health, social work, planning, environmental psychology, community development, geography). The complex nature of this work is best served through interdisciplinary perspectives, especially in light of increasing concerns related to natural processes impacted by human made activities [26,27]. The broader global issues of climate change, population growth, deforestation, natural resource exploitation, and widespread culture of consumerism impact ecological and social systems at the local level [26,28,29,30,31,32,33,34]. 

We position our research within two frameworks. First, we draw upon an ecological-social (eco-social) perspective that focuses on the multidimensionality of systems that interact with one another [26,31]. Our specific interest is in the relationship people living in four low-income neighborhoods across two large urban Midwestern cities have with their social, natural, and built environments. This includes multiple interacting ecological and social systems characterized by interdependence, diversity, vulnerability, and adaptation [26]. While the eco-social perspective frames our residents’ stories about “what is”, often pointing to ecological threats that can be human-made or natural, the focus group conversations also represent ideas about “what could be”. Secondly, we use an action-oriented, anti-oppressive environmental justice framework [30,35] as a way to frame residents’ conversations around identified power differentials in decision-making processes about their communities [36,37,38], individual agency to consider and/or engage in different individual behavioral changes or social, political, or collective processes [37,39,40,41], and perceived community-level capacity, efficacy, and engagement in systems-level changes [37,38,40,42,43,44]. Within an environmental justice framework, we specifically apply a Prepared, Engaged, or Leading Change (PEL) framework [37] and components of the nine-stage Community Readiness Model (CRM) [45,46] to describe individual engagement to improve their communities. We then discuss residents’ perspectives on broader community-level efficacy and capacity for change. 

## 3. Materials and Methods

The current research was part of a project focused on perceived risks and actual food safety concerns in four neighborhoods across two large urban cities in a Midwestern state with vacant lots potentially available for urban agriculture. The original research study with detailed sampling and coding methods has previously been published [47]. Data was obtained during four focus groups (one per neighborhood) [47]. Focus groups offer a less intimidating safe space to explore views, ideas, thoughts, and beliefs among large groups [48]. They offer participants the opportunity to explore individual-level impacts of issues at multiple scales and collectively share ideas for addressing them [48]. This can be especially useful when exploring topics that are not well-recognized, such as environmental stressors [49]. There are no agreed-upon rules for focus group samples across several disciplines involved with this study, though some research has said the ideal is 5–12 [50,51,52,53] while reported samples without much justification range from 1–20 [49]. Since our purpose was to engage residents from each of the four communities in an in-depth structured focus group discussion, we wanted to ensure our sample was reflective of the heterogenous communities of which they were a part [50]. Participants were recruited from four urban neighborhoods across two cities that had high poverty rates, land that was vacant and possibly available for food production gardens, and areas with the lowest median incomes in the cities. Researchers engaged with community leaders from civic associations, community agencies, and faith-based organizations who worked with low-income residents in each of the neighborhoods. Researchers explained the research purpose and worked with community leaders to identify appropriate locations for the focus groups that would be accessible, times focus groups would need to be held for ample participation, and parameters around participant requirements per IRB-approved protocol (e.g., living in the identified neighborhoods, at least 18 years of age). Most recruitment was done through flyers and word of mouth, which is the most common form of communication in each of these neighborhoods. They were distributed through our community liaisons and posted in public spaces, like the libraries in C1 and C2, where the focus groups were being held. We had to abide by library regulations for use of their free space, which was to allow the group to be open to anyone willing to participate. As researchers, we explained the IRB protocol and sampling procedures. We were concerned about participation rates even though we felt we had appropriate locations, incentives, and food available [51], so we overrecruited [54]. Knowing that incentives are an important reason for participation [50], researchers prepared themselves for potentially-larger focus group sizes by purchasing more grocery gift cards, having trained research assistants available to help with any group dynamic challenges that can come with larger sizes, assuring that the room had enough seating for people, making sure our stenographer could clearly see participant names, and detailing communication standards for each focus group (i.e., only one person at a time, raise hand, clearly show name, be patient if the stenographer needed clarification).

These focus groups were audio recorded, transcribed and reviewed, during which open coding was used to establish emergent themes in the data, as outlined by [55] in her recommendations for line-by-line open coding in qualitative research [47]. This thematic analysis approach involved the generation of initial codes using the exact words of participants, further organization into categories to yield descriptive themes, and finally, a comparison of similarities and differences between descriptive themes to result in higher level analytical themes [56], which were described and published [47]. After completing the scope of the interdisciplinary grant-funded work with people who have training in public health, microbiology, social work, veterinarian medicine, and soil science, two researchers recognized the richness of the data and re-analyzed it through an eco-social lens, resulting in the current study.

The first and second authors determined the questions that had not been analyzed in the previous publication and that would likely yield responses regarding the neighborhood environment. We first focused on the contextual and introductory question: “How would you describe your neighborhood to someone who does not live here?” To ensure re-immersion in the data, the first author re-read each of the transcripts, noting any responses that related to this research question as part of a theory-informed, or interpretive coding process [57,58]. While responses were analyzed within the context of this question and the above described environmental justice oriented eco-social perspective, data included in the present analysis also came from primary questions regarding perceptions of vacant lots and specific potential environmental agents that could be hazardous or unhealthy (e.g., litter or waste, industrial sites). The first author then shared the initial codes along with the descriptive and analytic themes that emerged, and associated quotes (to support these themes) with the second author (who had conducted the original analysis). Pseudonyms were used in place of the actual names of participants to protect the anonymity of, and to humanize them [59]. 

After re-reading the transcripts, it was apparent to the researchers that participants were expressing more about actions they were or were not taking, neighborhood processes, and sense of community. These themes were determined from the direct words of participants during each of the four conversations, reflecting a deeper conceptual interpretation about residents’ views and interactions with their natural, built, and social environments, within the context of the integrative ecological-social and environmental justice framework. The first author shared these themes with the co-authors, re-positioning the research questions to include individual and community efficacy and engagement. This interpretive approach allowed for further exploration of unique assumptions, ideologies and concepts that may help to shed light on and address distinct ecological and justice-oriented challenges participants were experiencing or witnessing [57] Consensus was reached on these themes upon review, after which a report was fully produced which includes both narrative and illustrative extracts from the transcripts to further illuminate the themes [57]. 

## 4. Results

### 4.1. Focus Group Participants

Our sample (n = 68) came from Community 1 (C1) (n = 27), Community 2 (C2) (n = 28), Community 3 (C3) (n = 8), and Community 4 (C4) (n = 5), with 66 participants completing a demographic survey. C1 and C2 are nested in the same city, and C3 and C4 are nested in another city. Detailed descriptive results have been previously published [47]; Table 1 provides sociodemographic information about the four broader communities where our research took place. Table 1 displays demographics by zip code (ZC) for each of the nine ZC in the study that represent four neighborhoods [60]. It is intended to provide researchers and practitioners information they likely have about their own communities of interest in order to compare and contrast our work with theirs, and it allows for readers to have context for comparison across all four neighborhoods for this study.

The average housing vacancy rate across C1 and C2 is 16.67%, compared to 22.1% for C3 and C4. Compared to the median household income of $50,700 in Franklin County [61] where C1 and C2 neighborhoods are located, four zip codes have median household incomes less than the average (ZC2, ZC3, ZC4, ZC5). ZC2 has the lowest median household income and is less than half of the county median household income. Correspondingly, ZC2 also has the greatest poverty level of the C1 and C2 zip codes. The median household income for Cuyahoga County where C3 and C4 are located, is $43,861 [61]. ZC6, ZC7, and ZC9 have median household incomes far less than the county level, while ZC8 has a substantially greater median household income than the county. ZC 8 has a poverty rate less than the state level of 16.7% [61]. 

The C1 sample (n = 27) had a greater percentage of Black residents than the Census data for the ZC where the neighborhood is located (33.3% compared to 19%). Of those who reported monthly income (17/27), all reported incomes lower than the neighborhood median household income. The sample has a high school or equivalent degree rate that is almost twice that of the neighborhood (51.9% compared to 28.5%), but much less than the neighborhood rate for undergraduate degrees (7.4% compared to 19.4%). Over 32% of participants from C2 (n = 28) identified as white compared to the neighborhood (58.8%), while 57.1% of the sample identified as Black, compared to 35.1%. The sample had a greater rate of individuals with a graduate degree (21.4%) compared to the neighborhood (8.3%). Of the 18 participants who reported monthly income, all reported less than the neighborhood median household income ($46,481). In C3, 87.5% of participants (n = 7) identified as Black, which is greater than the general neighborhood rate (71.1%). Twenty-five percent of the sample obtained a high school degree or equivalent, which is comparable to the neighborhood rate of 28.7%. Of those who reported household income (n = 5), all reported income less than the median household income. The racial makeup and education of the sample from C4 varies greatly from the general neighborhood, and this is due to a very small sample size (n = 4). All participants identified as Black, no one earned greater than an associate’s degree, and all who reported income (2/4) reported income less than the neighborhood median household income. 

### 4.2. Challenges in the Built, Natural, and Social Environment 

When asked to describe their communities, participants from each of the four neighborhoods discussed a range of observations they had of the built and natural environment, along with associated aspects of the social environment (see Table 2). From these observations and experiences, we identified five themes: (1) pollution, (2) abandoned buildings, low quality housing, and crime, (3) low food access and health, (4) trash and illegal dumping, and (5) lack of trees. While these themes were consistent across all focus groups, distinct differences were also determined across the groups.

Participants also described diversity within neighborhoods and different experiences depending on the block or street in that neighborhood. In addition, participants often stated their concern and then followed up with a litany of perceived causes or contributing factors to these issues, reflecting participants’ worldviews, experiences, and assumptions. Some provided reasons as to how these concerns impacted individual and community well-being.

#### 4.2.1. Pollution

One common theme across all groups was noise and air pollution. Participants described noise pollution related to traffic, loud music, and violence. The concern about the noise, especially coming from violent situations and disrespectful people is best reflected by Arielle, a resident in C4:

You just got tired of it. You trying to relax, all you hear is gunshots, or somebody arguing, somebody out there fighting […] I be like, don’t you get tired of that?

Participants in C1 and C2 described sewer smells and were concerned about nearby water treatment facilities. Car traffic and associated carbon emissions and exhaust from cars and city buses were often described as a cause of air pollution. Betty (C1) shared:

The carbon emissions are getting so bad that I’m cleaning off my walls constantly living right on a main street […] in front of a bus stop […] my air ducts stay dirty constantly. We’re changing the filters in my furnace constantly.

A participant from C2 specifically stated that acid rain was an issue due to the emissions from a major utility company serving the area. Someone from C3 expressed concerns about brownfield sites that exist in their Rust Belt community, and Leon (C2) similarly shared concerns and uncertainty about high rates of asthma and cancer risks:

[This neighborhood] has historically been one of the most industrial parts of town […] and there have been issues with the EPA with them [a local steel company] and with others, so it makes you nervous […] People worry about—different times when some of these stories are in the paper and people talk about the cancer rates being high, all these things, it’s hard to tell what’s real and what isn’t, you know, but there’s some concerns there.

In C2, residents also discussed the concern about the lakes and rivers where many people regularly went fishing for their food, citing concerns about contamination and safety. 

#### 4.2.2. Abandoned Buildings, Low Quality Housing, and Crime

Participants in three of the groups (C2, C3, C4) described the social and environmental ills associated with abandoned buildings, empty lots, abandoned housing, and rundown housing. Many residents were concerned about “squatters”, “the homeless”, and people using drugs who they observed residing in those spaces. Francesca (C2) shared:

People outside my neighborhood are coming in these houses sleeping. The slumlords don’t care. The houses get empty. You call them squatters […] There’s three guys in the house two doors from me that comes in there and sleeps nightly.

Juliet (C2) described a situation in which she thought the property owner was using the space for money laundering, which impacted people in the community because it increased the drug problems in the area. She stated:

We have one property owner who will rent to anybody for cash, and we know it’s a way of him laundering his drug money, and we have problems with people living in a garage or living in a shed, and their drug paraphernalia is out in the alley where kids are cutting across to go to school or out on the street where people are walking. That’s an issue.

Hudson (C3), who moved back into the community, focused on when this urban decay became apparent in the Rust Belt city, describing an additional outcome when food stores closed:

I think the decline probably began when I was very young, but you can see that after in the past 20 years, and the past 10 years and the past five years, there’s been more buildings boarded up. Business is leaving the area and things of that nature. So, [now] […] there’s not a lot of options in terms of where you can get food from.

Concern for children was voiced during conversations in C3 and C4. Participants described the need for more youth activities, sharing that several recreation centers had closed or reduced their hours, which is likely related to the economic and population decline of the area. Every neighborhood shared their concerns about crime, which included explicit details of robbery attempts and lengthy discussions about drug use, shootings, and overall violence.

#### 4.2.3. Low Food Access and Health 

Access to food proved challenging, with participants discussing high food prices and high cost of prescriptions that sometimes translated into decisions about whether to eat or take medication, higher rates of diabetes, and concerns about cancers. Participants in C3 were frustrated by the lack of full-service grocery stores, and the need to take public transportation to access affordable food. Many communities mentioned fast food restaurants and liquor stores. Demetrius (C3) shared his observations of liquor stores, and as a result, his distrust of the government, and what he perceived to be a form of social control and oppression:

It’s just that in every poor neighborhood, you have a liquor store or you have [store owners] that’s always selling liquor. It’s kind of like the government wants to keep the poor, poor. Which don’t make sense, but it seems like they killing us off by liquor. If you go to any poor neighborhood […] you can get liquor before you get food. That is ridiculous and that’s what most people choose […] You got all these hot spots.

Residents from C1 shared that, while they were pleased with the number of places where free food was provided (e.g., churches, food pantries), they simultaneously voiced a potential unintended consequence. Dan (C1) shared:

There are a lot of places you can get food, and that brings in a problem though too because the homeless will come down here and then they beg so it’s good and bad.

This statement reflected overall sentiments towards persons experiencing homelessness, despite the fact that several focus group participants disclosed their current or past status as being homeless.

C3 expressed fear and concern about food which they had learned from television, such as use of hormones and steroids and the impact on children’s health. Several participants stated they wish they could afford organic food because of health concerns. These messages made it harder to shop for food, especially on a limited income with few options. Adele (C3) said:

[I am] scared to eat anything, because every time you look up the news, they’re saying it’s going to kill you. There’s something wrong with this, something wrong with that. Well, we’re all going to die because we won’t be able to eat nothing […]. Bread going to kill you, something’s wrong with the bread. Vegetables, the pesticides. Can you tell you tell me what I’m supposed to eat because […] I’m only eating popcorn right now. That seems to be pretty safe so far.

Accessing food appeared to be a stressful endeavor for residents. Affordability and health concerns were made even more complex for some residents. C3 participants shared concerns about hospitals shutting down in the area, coping strategies using alcohol, and concerns about untreated mental illnesses.

#### 4.2.4. Trash and Illegal Dumping

Trash and illegal dumping were also identified as an environmental problem in each of the four communities. Residents discussed the nature of common trash and litter, which included cigarette butts, candy wrappers, food wrappers, rocks, glass, beer cans, food, and fast food waste. Participants shared how people from both within and outside the neighborhood threw garbage out of car windows. Others blamed the stores that used plastic bags. Evelyn (C1) said:

I get these plastic bags up on my patio […] it’s like Kroger and Aldi’s, all the little bags. I don’t know how they end up in the yard.

Emilia (C4) wondered why the city was not taking care of their neighborhood:

There’s always trash. It’s not often that the city actually coming down the street and actually sweeping the street, or whomever does picking up trash or things like that, outside of your regular garbage day.

Others were concerned about specific types of trash, like needles from people using drugs. Lucian (C1) explained what he saw and why it is a concern:

You see a lot of needles laying everywhere […] You might step on one. You can get just about anything from being stuck by a dirty needle, and people just throw them around like they’re nothing. It’s kind of crazy.

Gabriel (C4) shared a similar concern:

Empty vials or things like that. That’s an issue. Because little kids around, they going to get and play with it and who knows who’s HIV positive.

Participants were also concerned about people abusing drugs and their behaviors impacting their communities (e.g., theft, gun violence, crime, threats, child neglect). 

Focus group conversations included discussions about dead animals in the street, in empty lots, and in dumpsters, generally reporting who they felt were responsible. Isobel (C1) lives in a public housing complex and shared that residents whose culture includes “slaughtering animals in the house” often threw “the parts they don’t want in the trash. We find a lot of dead animals in our dumpster, goats, chickens, animal parts.” 

Residents were surprised to see ground hogs, skunks, and deer, though one participant said:

We’re intruding on their [deer] territory. All this was theirs before we came.

Betty (C1) exclaimed:

We’ve got a lot of rodents, raccoons, opossums, you name it. Beavers. I’ve been chased by all of them, yeah.

Many residents were especially angered by illegal dumping done by people outside of the community. One resident (C3) expressed he felt people used his neighborhood as a “dumping ground.” Sean (C2) stated:

They come from another neighborhood and driving [sic] their truck and throw trash on our property.

Another participant described looking out the window of public transportation while crossing major roads and seeing everything from tires to animals over the embankments, which he described as “weird dumping.”

Residents expressed potential environmental health hazards related to the trash. Nancy (C1) shared her concern:

Just trash laying by the dumpster and food with maggots and bugs crawling all over it, and rats and everything.

Betty (C1) added:

I’m looking at bugs. I’m looking at gook and smell—they [people] even defecate over there. They even defecated on my stoop.

She was also concerned about how “overflowing dumpsters” might impact the soil. Bevaun (C2) shared a concern related to how trash impacts mental health:

They might, by seeing the garbage, feel more depressed just by seeing it, it makes you feel depressed. So, I can only imagine just like seeing garbage all over. That would make me depressed, just seeing it.

#### 4.2.5. Lack of Trees

In every focus group, trees were discussed. Many residents surmised that the lack of trees in their neighborhood was due to removal by their respective cities, though the reasoning for the removal was not discussed. Several residents connected their observations of trees to childhood memories. Rosemarie (C2) shared:

When I was a young girl, there were fruit trees everywhere, and right now I’d say you’d be lucky if you found one mulberry bush now […] We had cherry trees, apple trees, pears, grapevines, and they’re pretty much extinct now.

In C3, Gabriel shared similar sentiments:

I’d like to see the days when the cherry trees was out, the grape vines that everybody had growing in their yard. When you was little, you’d go back in the yard and try to get off the little cherries off their trees and they’d run you out of the garden and everything […] You don’t see no cherry trees around here no more. You don’t see any of that stuff.

Beyond providing pollination, food, and a source of enjoyment for kids, people shared that trees were needed to improve the air quality in their communities and reduce noise from traffic.

### 4.3. Neighborhood Assets and Proposed Solutions

Although much of the conversation in the focus groups focused on the challenges that residents were facing in their neighborhoods, many residents identified neighborhood assets that existed in the community (Table 2). These included places where people could seek social support (e.g., families, churches, settlement house, wellness center), access food (e.g., food pantries, churches), grow food (e.g., gardens, urban farms), access health care (e.g., mobile health clinics, mental health clinics), seek housing services (e.g., homeless shelter, home repair programs, senior living centers), participate in community programs (e.g., library, recreation centers), and engage with public servants (e.g., community crime patrol, civic associations). In addition, residents shared various ideas and solutions for the neighborhood, many of which were directly connected to the assets. The focus was wide-ranging and included ideas like redeveloping land, planting trees, finding jobs for homeless individuals, making phone calls to people with perceived power, attending civic meetings, engaging with neighbors, working with safety patrol officers and police, working with one another to clean up houses, yards, and streets, and taking action to remove drug needles, trash, and dead animals.

## 5. Discussion

Urban environmental concerns are multifaceted and involve numerous private and public actors, individual, and systemic contexts [62]. The themes identified in the focus groups reflect interconnected eco-social issues common in research literature; descriptions are consistent with environmental justice frameworks that underscore historical processes that impact low-income communities and communities of color and involve potentially different competing interests (e.g., between city officials and residents) [63]. Disadvantaged areas are much more impacted by physical and social disorder, which involve neighborhood environmental aspects such as litter, graffiti, and substance abuse [64]. Pollution can be especially problematic. Noise pollution, which is linked to poor sleep and hypertension [65] is greater in areas with high unemployment, low education, poverty, and more renters than owners [66]. Neighborhoods with abandoned buildings, trash, rundown housing, drugs, gangs, and other social problems have higher levels of fear of feeling unsafe and poor mental health [67]. Issues that were reported in our study (noise, housing, air quality) influence mental health; residents who feel like they have little control over their surroundings reduce overall mental well-being [7]. Reported food access issues are consistent with much research [68,69]. Residents reporting illegal dumping are right to be concerned, as it compromises safety, environmental health, soil, the water table, plants, animals, and property values [70,71]. Additionally, as residents noted in their discussion of concerns about trees being removed, trees can be effective at improving air quality by reducing harmful air pollutants [72]. 

### 5.1. Individual Engagement and Efficacy

We considered what residents said about their role in community processes to improve the quality of life, what actions they were engaging in as actors in their communities, and ways in which leadership emerged from some of the communities. Here, we elaborate on the meanings and implications of these roles, in the context of research literature. Residents who are aware of the problems in their neighborhood, believe that the neighborhood is capable of dealing with these problems, have social ties to the neighborhood, and feel there is the capacity for strong leadership are more likely to be involved in neighborhood change efforts [40]. Psychological empowerment, or the relationship between ability, motivation, and social action is associated with leadership [73]. Homeownership, income, and length of residence has been found to be positively correlated to individual-level participation but not block-level participation, suggesting the potential of cohesive participation by poorer, less stable neighborhoods at a comparative level to wealthier, stable neighborhoods [41].

Though civic engagement is linked with increased empowerment [73], more research is needed on the influence of informal connections among neighbors on self-determination in low-income areas [74]. The PEL framework has potential for assessing the stage or level at which individuals in communities are “prepared, engaged, or leading change” [37]. We frame our discussion of individual engagement and self-efficacy to reflect these three levels in this section. 

### 5.2. Level 1: Preparation for Change 

In our analysis, people who spoke during the focus groups were aware of, and often focused on, problems their communities were facing. Environmental justice frameworks acknowledge that environmental issues happen over time [63]. Though persons most impacted are likely to have experienced layers of inequalities (e.g., poverty, structural racism), grassroots efforts to build power and “agency” with these voices is important to addressing environmental injustices [63]. Participants offered a range of explanations existed as to why problems were occurring and what they felt their role was in addressing those issues. Some seemed more resigned to the way things occurred in their community, expressing reasons why they stayed despite what they experienced or observed. This reflects the second stage (denial/resistance) and third stage (vague awareness) of the nine stage CRM [45,46]. For example, Demetrius (C3) shared:

You afford what you can afford. I mean, for now you got to deal with what you can deal with. I mean, that’s the way of life. If we can afford to be anywhere else, I’m quite sure we would move. But if we can’t, we got to deal with what we can deal with.

Several residents expressed their own lack of actions out of fear, apathy, or exhaustion (i.e., ‘I am just too tired to do anything’). Frederick said:

Anything can happen. Even needles. I don’t pick up needles up on the street.

In the lengthy discussion about dead animals in C2, Arielle expressed the concern and why she did not get involved:

They hit ’em, they leave ’em laying in the street. Don’t nobody pick ’em up, so you smell that for like two or three days before somebody decides they want to pick it up. Me personally, I don’t mess with dead animals. I’ll tell you in a minute, I’m not picking it up.

Some participants did not seem to believe their community could change, which is similar to a study showing that individuals expressing “learned helplessness and fatalism” were less likely to feel that change could occur [43]. Demetrius (C4) explained the nature of human behavior that he had observed in the community, leading him to be doubtful that a community garden would be seen positively in his community:

I understand you talking about people tearing down [abandoned buildings] and grow [a garden], and that’s cool […] But […] people going to do things out of spite, know what I’m saying, because I’m not saying everybody’s like that, but some people just because. That’s just like in life. If I see you doing something that I might like, and I’m like, wow, he’s doing this, and I might just hate on you. People do that. It’s the way of the world.

Arielle (C2) shared similar ideas to Demetrius (C4). She added:

I’m just saying, say you got a garden now, and maybe two or three families like, why they build that, we didn’t want no garden. They might like, okay, when everybody leaves, we gonna go over there and tear their tree down or dig it up or whatever they’re going to do to it, and then you come in you be like, ‘We just had a tree yesterday, where did it go?’

Others recognized their ability to control major community issues [43]. Emilia (C4) described a straightforward solution to the litter problem in the neighborhood:

If everyone just picked up pieces of trash that they saw, I don’t see it [trash in neighborhood] really being a problem.

During the discussion about trees removal, Betty (C1) enthusiastically shared that despite the trees being gone “the soil was still there”. She further expressed a willingness to address problems, sharing her strengths, uncertainty about how to address the problem, and rallying people around issues related to income inequality and failed promises of economic development opportunities in their community. Her motivation and sense that something could be done reflect her care and sense of ownership for the neighborhood [38,43], placing her at stage four (pre-planning) and stage five (preparation) within the CRM [45,46]. She said:

I mean, I’m willing to put in the work, and I don’t like too much hard work.· I’m more intellectual than I am hands on, but I’m willing to put in the work, be it on the phone, going to the meetings, whatever, to get it done.· I just don’t know who to go to get this done, because there’s so much worried about—our city recently has been more worried about bringing the $34 million in for that stadium, and the casino, than they are about the people that live around the area that have to survive whether we’re working there or not.

One resident exemplified evidence of the impact toxic stress had on her willingness to address community issues, expressing the desire to leave the community because it was too challenging and was impacting her well-being [7]. Narissa (C2) was exasperated by people who she felt were taking advantage of government programs and engaging in activities like selling drugs. Though she loved many neighbors, community gardens, and church members, her daily experiences were taking a toll on her.

I don’t know, it’s just real hard when you’re one of those people who gives to the whole community, and you see that stuff coming at you every day. […] It’s too stressful […] how long do you live in a place where you’re continually stressed out by the fact that you see little kids across the street that aren’t fed right so that their hands are shaking, or they’re in dumpsters at midnight when you know after school time they’re not properly fed […] there’s too many things that no matter what I and others do, it continues.

In C2, an underlying focus on faith impacted the way that people felt situations could improve, which was unique to that focus group. For example, Harrison shared:

It’s a lot of people out there needing help, and I know God brought me out of that situation, and I did thank God for what we did for me. And if he can do it for me, I know he can do it for other people, so that’s all I want to say.

### 5.3. Level 2: Engaging in Change

Many focus group participants identified ways they were already engaging in activities to address the identified environmental issues. Umbrosia (C2) encouraged people to buy locally, to “put the money back into the community” as a way to address economic issues in the neighborhood. Betty’s (C1) preferred method of action was calling people in positions of perceived power. She called the health department and Community Crime Patrol but didn’t “get much of a response”. Frustrated by the lack of support from her apartment manager regarding the overflowing trash at the nearby bus stop, she contacted the transit authority and attended meetings, but said:

I don’t get any help.

Her most recent action was to:

convince new residents in our building that this is part of the issue that they need to help me address, because we all live there. It’s up to us to do it.

Frederick (C3) expressed discontent with calling authorities about trash:

You call them and they don’t come out ‘til they feel like it. I get real tired of that.

Residents often worked with neighborhood block watch groups to address many of the problems associated with drugs in their communities. This method of surveillance could prove successful, as research has shown that having networks of neighbors policing the area can prevent some crime because potential offenders have concerns about being seen committing the crime [75]. Demetrius (C4) shared:

You see the needles there. We do the neighborhood watch and then we sweep it up, but other than that —That happens quite often, like two or three times a week […] but sometimes we just be like tired and don’t bother cleaning it up.

Narissa (C2) described efforts that she and other neighbors on their block watch have taken, which included documenting license plate numbers and documenting where individuals would go to:

Shoot up and do stuff all night long […] trying to sell it [drugs] out on the side.

She also described harassment she endured by a neighbor and “crack dealer” in which she says a police officer that cruises the area regularly showed concern by saying:

You need to move before we have to come get you in a body bag, because this is crazy, and it’s just that bad.

Though she continues to try and improve her neighborhood and “help them,” she said that it often results in retaliation.

Frederick (C1) shared how he was trying to help out by boarding up abandoned houses, picking up people’s trash, and helping his sister mow her lawn. Despite his frustrations, he continued his efforts to try to keep his area clean:

I boarded the houses a thousand times and these young guys go in there […] People come and throw garbage and stuff out on the street. Every morning I might pick up garbage and different bags and stuff. It’s how you going to keep it clean and keep it maintained.

Betty (C1) who shared she had been homeless, recognizes that some of the people who need money or who are homeless can help her with tasks outside of her apartment and she can help them identify community resources:

I’ll tell them, hey, you want this, you want that, here, I need my trash taken out. In the winter I need my sidewalk cleaned, my stairs cleaned off […] I’ll send them to where I know there’s resources.

She also shows a willingness to take action if she has any problems with people, sharing that she calls the community police if the homeless “are becoming a problem.”

### 5.4. Level 3: Leading Change

While community relationships are very important, successful collective efficacy requires equitable agency when establishing formal state and community leaders while addressing economic and racial inequality [76]. During the focus groups, several people emerged as leaders. The idea that leadership may come from the community itself is important. Perceived strength of neighborhood leadership, a dimension of community capacity, was the strongest predictor of neighborhood engagement [40]. 

Betty (C1) energetically shared how she tried to engage neighbors to take their fight for trees as a proposed solution to carbon emissions to city leaders:

I’ve been trying to talk some of my neighbors into, let’s see if we can get city hall to help us plant more trees.

Oliver (C2) acknowledged that it would be easier to be apathetic and not care about the neighborhood, but he chooses to lead by example, despite concerns about unintended consequences of leadership (e.g., being targeted by people who don’t like what he is doing):

I guess my opinion is, is that I’ve lived here for 12 years, and when you start to care, it’s like the neighborhood will test you, you know. I got my house cleaned up, and I had neighbors say, “Don’t fix up your house, you’re just drawing attention to yourself”. Well, I care. I live here and this is how I want to live, and, you know […] people will take from you if they think that they can. […] Until we say this is not acceptable that I live next door to this, or that you’re here and acting this way and we don’t say anything because we’re afraid—if you stand up for yourself, you are probably going to get targeted, but until we stand up for ourselves, nothing is going to change. So we have to start doing that.

Bevaun (C2) had similar beliefs, and was of the opinion that working hard will eventually wear down those who are trying to destroy that work:

Just got to keep doing it [gardening]. Just because they do something to it, doesn’t mean you should stop. If you keep doing it, they’re just going to get tired. So sooner or later, they’re going to—they destroy your garden, plant it back. They’ll get discouraged when you just keep coming back. Keep coming back.

### 5.5. Community Engagement/Efficacy

Though many participants mentioned their current efforts to improve their community and willingness to take action, residents also acknowledged that they needed broader support from existing systems in which decisions were being made on behalf of their neighborhoods. A community’s capacity for change is reflected in residents’ sense of community and collective efficacy, or the idea that neighbors have shared expectations, trust, and take action together [38,43,76]. Participants also shared challenges they had experienced with civic engagement, discussed underlying processes that existed to improve their community, and described how their sense of community played a role in how well they felt their community was equipped to address issues. 

Betty (C1) identified city council leaders as an important group to engage for several issues that could impact relevant neighborhood issues, but also identified a longer term opportunity to influence the structure of council (i.e., having “neighborhood delegations”) so that it would be more representative of unique voices from different neighborhoods across the city.

Juliet (C2) was frustrated about “slumlords”:

Then she [property owner with tenants abusing drugs] came begging to the neighborhood group, help [her] get rid of them.

(Juliet, C2)

Juliet then shared how they helped the property owner:

We have awesome police down here, community crime patrol, and other folks who would spend months writing down license plates and doing all this other stuff to give that to the police so we could get rid of them.

Juliet chose to engage an individual (the “slumlord”) with whom she was angry and frustrated, accessed the existing trained officials who uphold their duty to protect people, their network of concerned and engaged neighbors, and a non-profit crime prevention group. Juliet’s efforts were successful:

Now I’m not scared to take my dog in the backyard at midnight to do her business, so that’s good.

Betty (C1) also mentioned the importance of the Community Crime Patrol and police. She identified that business owners were interested in having residents and customers feel safe:

So even though many “don’t all live down here […] they care about the community […] If they see one of the ladies being harassed, maybe a homeless person asking for change, they’ll step out from their businesses and stop it.

Her sense of community reflects research that is safe and has an appropriate level of resources and services [77].

C4 participants expressed dissatisfaction and uncertainty about the willingness or capacity of city services to support environmental efforts with them. Demetrius explained:

I think the trash or whoever, [the city] supposed to— they should come by and clean that up. At least once or twice. I don’t see them never do it, which we don’t never have that come by.

Frederick (C3) did not trust that contacting the police made a difference in his community when his observations were that the police did not take appropriate actions:

I called the police about abandoned houses, but they go turn the knob on the door—I watched them—turn the knob on the door and walk away, get in the car. I called them back, I say, you didn’t even go in the house […] What good is calling the police? They don’t do nothing […] They give you a reference number, it don’t mean nothing. They get to it maybe a month later when there are rats or something. It’s just pathetic.

Participants from C1 disclosed their concerns about crime, but for the most part, felt connected to their neighbors. Betty said:

I give a helping hand so you can get a leg up and get on down the road

Dan (C1) said:

We do take care of our own people.

Ann (C1) said that as a senior, she feels protected:

There have been a couple incidents that they tried to harm us, you know, but we stick together.

C2 residents similarly shared that “people look out for each other” (Narissa). Arlena (C2) discussed the diversity of the community, in comparison to where she was raised, and how neighbors help one another:

I grew up in the projects […] And what’s different as growing up—well, as a child we didn’t know about prejudice, but black people stayed by theirself [sic], and the white people stayed to theirself [sic]. And for this neighborhood, for me coming to it as an adult, even as growing up as different neighbors, I’ve learned from this neighborhood, very loving, is the diversity that we have […] No matter who you are, what you have done, we’re all people together […] if you need help, if I need help, we have each other.

On the other hand, C3 and C4 conversations tended to focus on fears residents shared about others, often describing concerns about starting gardens and people intentionally contaminating them or ruining them. Overall, these sentiments may point to low social structure and solidarity [64].

#### 5.5.1. Study Limitations 

Each community focus group discussion was unique to the circumstances in their neighborhood, but also to the number of people in the focus group and the tone and dynamics of the focus groups themselves. The conversations in C1 and C2 around assets and proposed solutions were much more involved. This was likely due to the larger number of people in those groups and the location of those focus groups. Both were held in public libraries that have rules that our focus groups had to be open to the public’s participation, unless people did not meet our IRB requirements of being younger than 18, not living in the identified zip codes, or arriving after we started our focus groups. Since we had grant funding for grocery gift card incentives, we were financially able to accommodate larger focus group sizes, though it was not feasible with time constraints of the grant to host more than four focus groups [50]. The facilitator and assistants were comfortable and trained in working with diverse groups of varying sizes and anticipated this challenge due to the library rules. Furthermore, we did not want to collect identifying information ahead of time through a phone call or email RSVP, as per our IRB-approved protocol. It was also important to consider location for each focus group so that it was accessible and available at times when people could participate. People in C1 and C2 were used to attending meetings at the library about neighborhood issues, and many seemed to know each other and feel comfortable expressing their thoughts with our research team. Our team had stronger community connections to leaders in C1 and C2 and were easily able to rely on them for widespread recruitment and communication about our focus groups. IRB human subjects research protocol does not require a focus group participant to contribute to the discussion. However, even though we had large sample sizes in C1 and C2, and some studies have shown that our sample size would be considered less than ideal for fear that people would not feel comfortable participating [50], we did not have that experience in C1 and C2. In these communities, people seemed willing to talk about their community’s issues, share their perspectives, and trust one another. In C1, 74% of people contributed to the focus group conversation, and in C2, 86% of people contributed to the focus group conversation All completed the surveys. 

Our C3 and C4 meetings were held in a central location at a college student union off of a bus line. While we coordinated with two key liaisons and their network, the attendance was much lower. We asked participants why they thought others would not come and there was generally a lack of trust of people outside the community, the weather was rainy, and the cohesiveness of the communities was much less than C1 and C2. In addition, while the location was easily accessible, we recognize that having a meeting on a college campus may not feel accessible for persons living in C3 and C4 where college education rates are very low. The C3 focus group, specifically, was heavily dominated by the problems of the community. People were less inclined to discuss what they appreciated about their community or ways to improve it. We were more limited on time for the C3 group because our C4 focus group was being held immediately afterwards and we started late to wait for people arriving from public transportation.

#### 5.5.2. Policy Recommendations 

Though the original intent of this research was not related to policy, our purpose was to elevate the lived experiences of residents living in communities that are facing innumerable challenges of poverty, crime, trash accumulation, illegal dumping, health concerns, and poor food access. Our analysis shows that these voices and their perceptions are crucial to efforts that can address environmental health issues and improve the quality of life for all residents there. These conversations were impactful for residents and have contributed to many new programs and efforts for C1 and C2. We are less aware of changes in C3 and C4, but this may be due to stronger relationships with community members in C1 and C2, the location of researchers in the city where C1 and C2 are located, and continued community-engaged research in C1 and C2. 

Residents were deeply concerned about litter, illegal dumping, and homelessness in C1. Since the focus groups, the chamber of commerce worked with the city to obtain funds to support several resident-driven efforts to address these issues. This includes residents who work together in teams to clean-up assigned areas throughout the year. They are given supplies and special t-shirts and compete to remove the most litter from their neighborhood block. These citizen participation efforts are impactful, as neighbors and children see people they know picking up trash, which can have a positive psychological impact on behavior. In addition, it builds camaraderie and encourages people to care for the natural and built environment. The city is also supporting an initiative to pay persons who are chronically homeless to beautify and clean up areas along the commercial corridor that is still largely vacant. The clean-up efforts and re-investment by the city are part of a larger city plan to expand this corridor and attract businesses for economic development purposes. Residents in C1 have also created online Facebook groups to track illegal dumping in their communities and encouraged the use of the city’s online service report system to bring attention to the issues and have overflowing dumpsters in alleys immediately removed.

Throughout the city where C1 and C2 are located, efforts to increase citizen participation in democratic processes have occurred. Citizens can serve on area commissions and are encouraged to attend meetings to voice their concerns. Several neighborhood civic groups exist to discuss issues before bringing them to government officials or the area commission. People are invited through paper flyers in mailboxes, door-to-door canvassing, and online social media groups. A local agency hosts a neighborhood leadership academy to help residents in these low-income communities develop their leadership skills and understand democratic processes. A progressive political group has organized efforts to recruit, train, and support residents from these neighborhoods to participate in coalition-building around progressive values that are intended to improve the quality of life for their neighborhoods. This includes supporting candidates for serving as representatives on the Democratic Party Central Committee of the county.

Lastly, since our focus group conversations, some of the residents from C1 and C2 have worked together to improve healthy food access. In C1, the urban farm has expanded to include perennial fruits and berries along with their annual vegetables and herbs. They have received federal funding to expand their food production space and implement a community-supported agriculture (CSA) program for residents in their neighborhood. The CSA delivers weekly produce grown on previously-vacant lots and works in coordination with programs serving low-income households. Over 140 households have participated since 2016. Residents in C1 and C2 participated in neighborhood meetings to help inform a local food action plan that was adopted by the city and county. In C2, a focus group member helped lead efforts to build an urban fruit tree forest and has led efforts to bring food-related businesses and restaurants to their neighborhood. C2 focus group members worked with a local church, community development agency, private and public sponsors, and the regional foodbank to transform a former drive-through liquor store into a free fresh produce market. A community center also hosts free weekly community meals that are highly attended and has a pay-as-you-can restaurant. Both areas have seen increased businesses. 

Information from our focus groups are extremely valuable to stakeholders in the community and policymakers who likely reside outside of the community. Our methodology of working with community leaders in C1 and C2, in particular, has allowed for conversations that started in those two-hour meetings to continue. Researchers involved in this study continue to conduct work in C1 and C2 and people still reference those focus groups at the libraries. Our analysis shows that resident voices and efforts are an important part of the solution. It is recommended that researchers share their analysis with policymakers and provide briefings back to communities to continue conversations and ensure involvement by residents who are most impacted by the challenges and can be important change agents.

## 6. Conclusions

Approaching individual or collective efforts to improve the built, natural, and social environments needs a multi-pronged approach that starts with residents’ voices. They know best the issues that are most concerning in the community. This study focused on the lived experiences of residents living in low-income urban neighborhoods in their own word. They focused on five specific challenges in their community: Pollution, abandoned buildings with associated crime, low food access and health concerns, trash and illegal dumping, and lack of trees. Further analysis showed that within each community there are individuals who are engaged, potential leaders that may benefit from support and training, and others who can easily identify barriers that could be targeted for efforts by trustworthy groups. Two of the communities (C1 and C2) in our study seemed to have a greater sense of community, awareness of specific resources, opportunities for impact, and potential grassroots leadership, while C3 and C4 seemed to have little trust in political systems and civic structures. This study was not intentionally targeted for policy change. However, the focus groups served as spaces where conversations led to action. Policies have been enacted and programs have been created, in part due to leaders in the focus groups engaging with community members to address their challenges. While these results may not be overly generalizable, knowing where to start comes from listening to the people experiencing environmental injustices and recognizing that “A land ethic begins with knowing and loving the land, and committing to citizenship within it,” (summarizing Aldo Leopold) [78].

## Figures and Tables

**Table 1:** Community Characteristics.

	Community 1 (ZC1, ZC2, ZC3)			Community 2 (ZC4, ZC5)		Community 3 (ZC6, ZC7)		Community 4 (ZC8, ZC9)	
	ZC1	ZC2	ZC3	ZC4	ZC5	ZC6	ZC7	ZC8	ZC9
* Population	12,790	4617	27,366	21,864	45,144	20,136	23,073	40,438	22,640
	%	%	%	%	%	%	%	%	%
* Race White Black American Indian Asian Two or more races	73.7 18.4 0.3 3.4 2.8	73.2 18.0 0.5 1.2 5.1	72.1 20.5 0.4 1.6 3.5	50.3 43.9 0.3 1.2 3.5	67.3 26.2 0.3 1.3 3.2	13.8 83.8 0.2 0.1 1.6	5.1 92.9 0.2 0.2 1.5	57.6 36.7 0.1 2.5 2.5	1.7 96.7 0.1 0.1 1.2
* Ethnicity Hispanic or Latino	4.1	4.1	4.4	2.3	3.4	1.3	0.9	2.1	1.0
* Housing Vacant units	11.5	25.9	15.4	17.56	13.0	22.8	31.9	12.2	21.5
** Education HS graduate/equiv. Some college Associate’s degree Bachelor’s degree Grad/prof degree	9.3 12.6 3.7 43.3 26.4	33.8 12.7 5.4 10.8 1.9	42.4 20.0 5.3 4.1 1.8	27.2 16.8 5.4 23.9 13.9	40.6 20.9 6.2 8.6 2.7	33.9 25.6 9.6 7.1 2.8	36.9 27.6 6.7 7.9 5.1	15.2 18.3 6.9 25.6 27.9	39.3 22.3 5.9 4.0 2.3
** Unemployed	3.3	8.0	7.7	10.3	10.9	21.0	19.2	6.0	34.0
** Median household income	$57,752	$23,802	$32,724	$50,660	$42,302	$21,468	$22,147	$61,044	$14,646
** % below poverty	20.8	37.2	35.5	24.5	24.4	40.5	38.7	16.5	58.3

Source: * 2010 Census, ** 2017 American Community Survey.

**Table 2:** Focus Group Findings.

	Observed Built, Natural, Neighborhood Environmental Concerns	Perceived Causes of Built, Natural, Neighborhood Environmental Concerns	Neighborhood Assets	Proposed Ideas and Solutions for Neighborhood
**Community 1**	Sewer Smell Noise Pollution Air Pollution Dirty Air Ducts Empty Lots Abandoned Buildings Household Trash and Overflowing Dumpsters Needles on Street, Lots, and Sidewalks Dead Animals in Dumpsters Homelessness No Trees	← Water treatment plant ← Cars, Expressway ← Cars, Overflowing Dumpsters, Dead Animals ← Carbon Emissions from Cars None Stated None Stated ← People littering, Grocery stores that use plastic bags, City for not cleaning up ← People using drugs ← People slaughtering them for food ← Abandoned buildings, Easy access to services ←City removed them	Senior Living Center Settlement House Urban Farm Food Pantries Mobile Health Clinics Churches Offering Food Hospital w/ Free Wellness Classes City Program for Reporting Nuisance Houses Ministries Serving Homeless and Low-Income Mental Health Services Free Health Clinics Rich Soil for Growing Food Online City Services Program Neighborhood Liaison Centers w/ City Community Crime Patrol Active Civic Association Sense of Community Library	Redevelop brownfields Create more green areas for kids Plant trees Jobs for Homeless Call health department Organize neighbors Work to change City Council Configuration to be more neighborhood-centered Talk to businessowners about any problems Attend Civic Association Meetings Find information at Library
**Community 2**	Acid rain Air pollution Noise pollution Soil concerns Unclean lakes or rivers Crime (robbery, gun violence) Illegal dumping Homelessness Abandoned or rundown housing Drugs Drug paraphernalia High cancer rates Water run-off Lack of trees	←Companies trying to repeal Clean Air Act ← Car exhaust, water treatment facility ←Cars, loud music ←Buried trash, acid rain, bricks, rocks None Stated ←Drugs, squatters, distrustful people ←People outside community ←Slumlords, system of money laundering None Stated ←Squatters, careless people None Stated ← Pavement, poor planning for drainage ←City removed them	Diversity Various gardens Free store Homeless shelters Home repair programs Sense of community Community development organization Church Library Church food pantry Faith University Extension Programs Community leadership Neighborhood association	More participation in gardens Engage with neighbors Work with community crime patrol Buy local Clean up your house/yard and set an example Keep going Stand up for yourself Trust faith Help property owners (also slumlords) to remove problem tenants Communicate w/ Police
**Community 3**	Abandoned buildings, gas stations Lead Paint Limited healthy food access Robbery Garbage and illegal dumping Idle kids Asthma Cancer, diabetes Mental health issues Brownfields Needles everywhere Lack of trees Alcoholism	←Businesses leaving, economic decline ←Housing stock deteriorating and paint flakes off ←Lack of grocery stores, need transportation to access, food prices, medication costs None stated ←People throwing garbage out of vehicles, People from inside and outside neighborhood ←Lack of activities ←Car exhaust, something in the empty lots None Stated ←Medication costs limiting ability to get prescription filled or re-filled None stated None stated None Stated ←Liquor store hot spots, lack of mental health care	Library Free food at library for kids Food pantries	Summer jobs for kids Work with neighbors to pick up trash
**Community 4**	Loud music Bad kids Garbage everywhere Bottles, needles, glass Dead Animals Gun violence Diabetes and health issues Drug issues	← Disrespectful people ←Recreation centers closed, reduction of recreation center hours ←People throwing fast food out window, No street sweeping, Inconsistent garbage removal from city ←People using drugs or alcohol ←People not cleaning them up after hitting them with car None stated ←Stress and diet None stated	Public transportation Family nearby City councilman living in neighborhood Recreation centers Anything free Library Food pantries Food bank	Opportunities for young kids to learn gardening Free fresh fruit baskets More activities for kids and young people Places for kids to get out their frustrations Involving kids in community events Test soil Clean up the streets Individuals pick-up own trash Consistent dead animal removal Work w/ Neighborhood Watch to remove needles

Language reflects that of the participants.
